# Use of Biodegradable, Chitosan-Based Nanoparticles in the Treatment of Alzheimer’s Disease

**DOI:** 10.3390/molecules25204866

**Published:** 2020-10-21

**Authors:** Eniko Manek, Ferenc Darvas, Georg A. Petroianu

**Affiliations:** 1 College of Medicine & Health Sciences, Khalifa University, Abu Dhabi POB 12 77 88, UAE; eniko.manek@ku.ac.ae; 2 Herbert Wertheim College of Medicine, Florida International University, 11200 SW 8th St, Miami, FL 33199, USA; ferenc.darvas@fiu.edu

**Keywords:** Alzheimer’s disease, polymer nanoparticle, biodegradable, chitosan, blood-brain barrier, CNS delivery, amyloid-β

## Abstract

Alzheimer’s disease (AD) is a progressive neurodegenerative disorder that affects more than 24 million people worldwide and represents an immense medical, social and economic burden. While a vast array of active pharmaceutical ingredients (API) is available for the prevention and possibly treatment of AD, applicability is limited by the selective nature of the blood-brain barrier (BBB) as well as by their severe peripheral side effects. A promising solution to these problems is the incorporation of anti-Alzheimer drugs in polymeric nanoparticles (NPs). However, while several polymeric NPs are nontoxic and biocompatible, many of them are not biodegradable and thus not appropriate for CNS-targeting. Among polymeric nanocarriers, chitosan-based NPs emerge as biodegradable yet stable vehicles for the delivery of CNS medications. Furthermore, due to their mucoadhesive character and intrinsic bioactivity, chitosan NPs can not only promote brain penetration of drugs via the olfactory route, but also act as anti-Alzheimer therapeutics themselves. Here we review how chitosan-based NPs could be used to address current challenges in the treatment of AD; with a specific focus on the enhancement of blood-brain barrier penetration of anti-Alzheimer drugs and on the reduction of their peripheral side effects.

## 1. Introduction

Alzheimer’s disease (AD) is a progressive neurodegenerative disorder that affects over 24 million people worldwide; representing an immense medical, social and economic burden [1]. The pathology of AD includes memory loss, spatial disorientation and pronounced decline of intellectual capacities due to neuronal cell loss in higher brain centers [2,3]. The etiology of AD is not yet fully understood and several factors are currently considered to play a role in its pathogeneses, including:

- increased inflammatory response
- accumulation of reactive oxygen species (ROS)
- deposition of amyloid-beta (Aβ) protein
- acetylcholine (ACh) deficiency
- deposition of neurofibrillary tangles (NFTs) of tau proteins
- metal ion dynamic equilibrium disorder
- lack of neurotropic factors, etc. [4,5,6,7].

In addition to the abovementioned causes, genetic predisposition, hormonal disorders, mitochondrial dysfunction and calcium toxicity may also contribute to the development and progression of AD [8]. Recently a wide variety of active pharmaceutical ingredients (API) were suggested to treat the symptoms of Alzheimer’s disease, including cholinesterase and phosphodiesterase inhibitors [9,10], non-steroidal anti-inflammatory (NSAID) drugs [10], antioxidants, tau hyperphosphorylation (e.g., GSK3 serine-threonine kinase inhibitors, such as thiazolidinediones) [11] and intracellular NFTs inhibitors (e.g., methylene blue) [12], estrogenic hormones [13], insulin resistance medications, metal chelators [14], vitamins [15], stem cells [16] and neurotrophins (e.g., BDNF, brain-derived neurotropic factor) [17].

One of the main obstacles that inhibit the development of effective medications for the prevention and treatment of AD is the selective nature of the blood-brain barrier (BBB), which prevents brain penetration of a large number of central nervous system (CNS) drugs [14,15,18]. As a consequence, only less than 5% of APIs are able enter the brain by passive diffusion [19]; mainly molecules with low molecular weight (Mw), lipid solubility and a small, positive charge [1,20,21,22,23,24,25]. The other major obstacle in the systemic treatment of brain diseases is that many CNS drugs have to be administered at high doses to reach satisfactory therapeutic efficacy, which results in severe peripheral side-effects [26,27]. In an attempt to overcome limitations represented by the BBB, various colloidal delivery systems have been developed in the last two decades that exploit the benefits of particle size reduction, such as polymeric NPs, liposomes, metal NP-based carriers, solid-lipid NPs, cubosomes and emulsions [28]. Among these nanocarriers, polymeric nanoparticles (NP) seem to be particularly suitable for the enhancement of drug pharmacokinetics, as they are able to mask the BBB transport limiting physicochemical properties of API molecules [24]. Polymeric nanocarriers are also ideal tools to reduce peripheral side effects, either by facilitating sustained drug discharge [29,30,31] or by enabling controlled API release via the incorporation of stimuli-responsive building blocks [32]. Polymeric carriers can bind drugs either covalently and non-covalently, as well as are able to carry both hydrophilic and hydrophobic therapeutic compounds [33]. Furthermore, polymer-based NPs exhibit an array of additional benefits, such high stability, facile production, high encapsulation efficacy, possibility for sterilization, easy modification with targeting ligands and multifunctional nature [19]. However, while there is a wide variety of polymeric NPs that are biocompatible, nontoxic, non-immunogenic, non-thrombogenic and stable in blood, many of them are not biodegradable and thus not suited for brain delivery [34].

**Figure 1:**
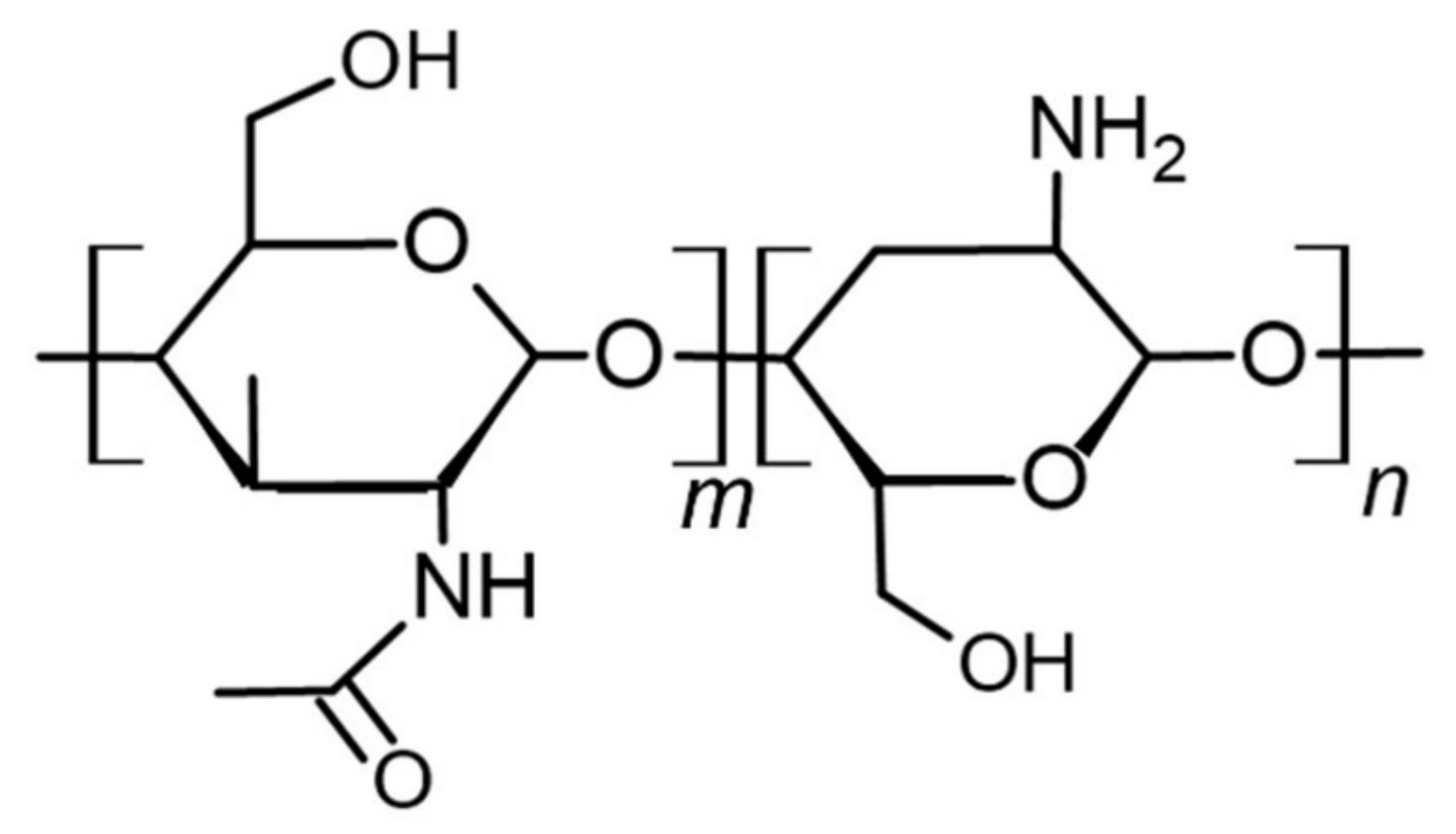
Chemical structure of chitosan.

Among polymeric nanocarriers, chitosan-based NPs emerge as biodegradable yet stable vehicles for the delivery of CNS medications. Chitosan is a natural, linear amino-polysaccharide that comprises of glucosamine and N-acetylglucosamine units (Figure 1). Due to its free amine groups, chitosan can be easily forged to NPs either by crosslinking or by exploiting its tendency to spontaneous self-assembly [35]. 

**Scheme 1:**
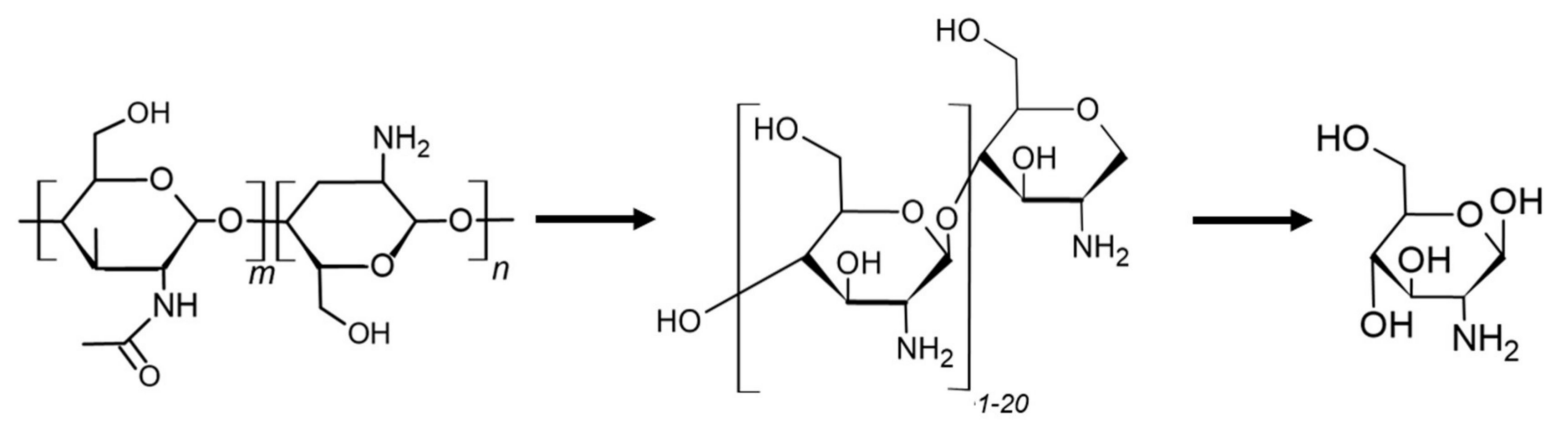
The enzymatic degradation of chitosan.

Chitosan’s advantageous properties are manifold, including biocompatibility, low toxicity, low immunogenicity, flexibility in surface modification and antibacterial activity [36,37,38,39]. Unlike most polymers, chitosan also shows cationic and mucoadhesive character, which is particularly suitable for enhancing cellular uptake by ionic interactions as well as for promoting penetration of drugs via mucous membranes [36,38]. Due to its D-glucosamine groups—which make chitosan structurally similar to sugars that are often used as cryoprotectants—chitosan can also serve as a cryoprotectant agent during the lyophilization of anti-Alzheimer therapeutics [40]. Owing to these beneficial characteristics, chitosan is a widely reported nanocarrier of a vast array of drug molecules, genes as well as proteins, and it has also been extensively studied as a material for brain scaffolds and spinal cord implants [39]. In view of CNS delivery, one of the most important attributes of chitosan is its biodegradability by different human enzymes as well as bacterial enzymes present in the gut flora; including chitosanase, chitinase, chitin deacetylase, β-*N*-acetylhexosamidinase and collagenase, as well as lysozymes, lipases and proteases. The major product of the enzymatic degradation of chitosan involving deacetylation and depolymerization processes is chitosan oligosaccharide (COS), which can be further transferred to D-glucosamine units (Scheme 1) [41]. All of these degradation products proved to be nontoxic, non-immunogenic and non-carcinogenic [39]. In addition, chitosan itself shows a notably high LD_50_ value of 16 g/kg, which approximately equals to that of salt or sugar [34]. From the production point of view, the advantage of chitosan NPs is that they can be manufactured easily and rapidly, and both the material and production costs are low [42]. On the flip side, limited water solubility of most chitosan forms can obstruct the development of nano-pharmaceuticals. However, chitosan is soluble in dilute aqueous acidic media (pH < 6.5) [43], which enables its engineering to NPs using a wide variety of techniques such as emulsification, reverse micellization, ionic gelation and desolvation [31,34].

The objective of present manuscript is to review how chitosan-based nanoparticles can be used to overcome current challenges in the treatment of Alzheimer’s disease; with a specific focus on the enhancement of blood-brain barrier penetration of therapeutic agents and on the reduction of peripheral side effects.

## 2. Use of Chitosan Nanoparticles in the Treatment of Alzheimer’s Disease

### 2.1. Chitosan - Acetylcholinesterase Inhibitor Nanoparticles

**Figure 2:**
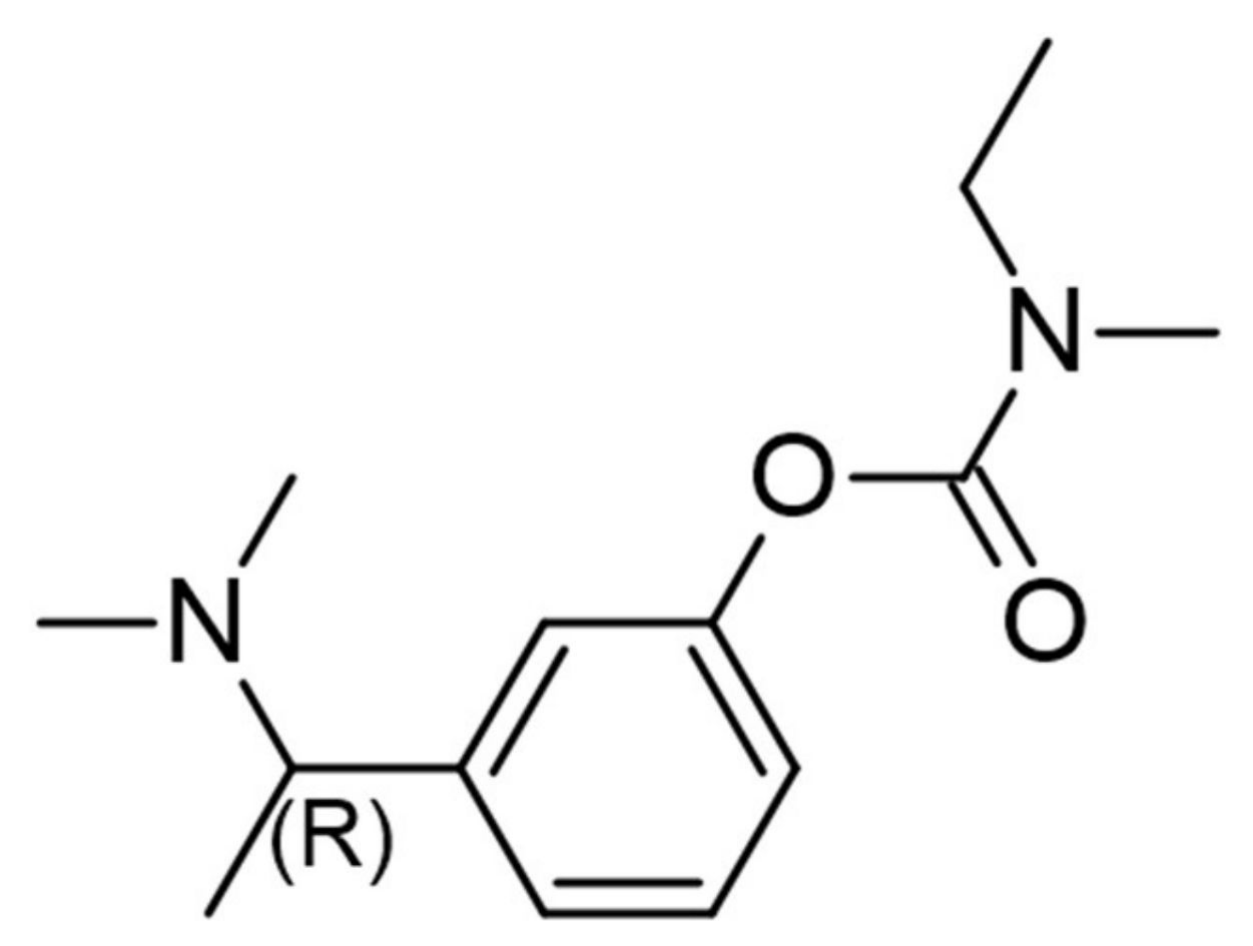
Chemical structure of rivastigmine.

Rivastigmine (RT) ([3-[(1*S*)-1-(dimethylamino)ethyl]phenyl] *N*-ethyl-*N*-methylcarbamate) (Figure 2) is a reversible anticholinesterase inhibitor widely used to treat mild and moderate cases of AD [44]. Rivastigmine was reported to possess both better efficacy and safety than tacrine [39,40]. Further benefit of rivastigmine compared to other anticholinesterases is its ability to inhibit both acetylcholinesterase and butyrylcholinesterase (BChE) [45]. On the other hand, rivastigmine is characterized by a very short half-life (1.5 h) [45], a relatively low absolute bioavailability (approximately 36% after a 3 mg dose) [20] and limited BBB penetration due to its hydrophilic nature. Furthermore, in conventional formulations rivastigmine is associated with cholinergically-mediated, mainly gastrointestinal side effects; such as abdominal pain, nausea, dyspepsia and vomiting [29,32,42]. While transdermal patches were reported to successfully reduce gastrointestinal adverse effects of rivastigmine, they can exhibit other disadvantages such as limited diffusion of therapeutic molecules via the skin barrier, skin irritation, loss of patches from the skin, or the lack of BBB transfer promoting ability [46].

With the aim of developing an optimized, biodegradable and biocompatible RT formulation for brain-targeting without the use of organic solvents, Nagpal et al. encapsulated the API in chitosan NPs by using the ionotropic gelation method [44]. The optimized formulation was evaluated by behavioral, biochemical and maximum tolerated dose (MTD) studies on male Swiss albino mice and male Wistar rats showing symptoms of amnesia induced by scopolamine. The in vivo studies confirmed that encapsulation of RT in polysorbate 80-coated chitosan nanocarriers considerably reduced the biochemical and hematological adverse effects of the drug and notably increased its MTD (from 1.5 mg/kg to 2 mg/kg). Both findings demonstrated that loading RT into polysorbate-modified chitosan nanocarriers can effectively enhance its safety. Aside improving drug safety, encapsulation of RT in coated chitosan NPs largely improved both its anti-amnesic effect and its BChE inhibiting efficacy. Interestingly, there was no significant difference between the effects of the free and the non-coated NP incorporated RT on amnesia and BChE.

Similarly to Nagpal, Khemariya and coworkers also prepared chitosan-RT NPs by the ionotropic gelation method [47]. The biodistribution of rivastigmine after intravenous (i.v.) administration in the form of nanoparticles (dose not specified) was analyzed by HPLC measurements in mice. It was reported that encapsulation in chitosan NPs increased the brain concentration of RT by 56% (52.8 ± 3.4 ng/mL) compared to the free form of the drug (38.8 ± 3.7 ng/mL). It was also found that brain deposition of RT could be even more drastically enhanced by loading the drug in polysorbate 80 coated chitosan nanocarriers, in which case RT brain concentration was elevated by 330% (168.1 ± 10.00 ng/mL).

Fazil et al. prepared chitosan-RT NPs for intranasal administration, also by ionotropic gelation, with the aim of avoiding first pass metabolism of the drug and limiting its peripheral side effects by avoiding distribution to non-targeted sites [38]. Due to the mucoadhesive nature of chitosan, improved nasal residence and subsequent increased brain uptake were also expected by the authors. It was demonstrated that encapsulation in chitosan NPs provided significant increase in the cumulative percentage of drug permeated via the nasal mucosa (70.1%) compared to its free form (20.3%). Biodistribution of chitosan-RT NPs was investigated on Wistar rats (either sex) by confocal laser microscopy after dyeing the nanocarriers with rhodamine-123. The microscopic studies confirmed that the loaded NPs accumulated in the brain rather than in the liver or lung after intranasal (i.n.) administration. RT brain/blood ratio measurements showed that drug concentration after i.n. administration of nanocarriers was remarkably higher (1.712) than either in case of intranasal (0.790) or intravenous (0.235) dosage of free RT. In accordance, brain concentration of RT for NP-assisted delivery (966.0 ± 20.7 ng/mL; t_max_ 60 min) greatly exceeded the brain concentration of both the intranasally (387.0 ± 29.5 ng/mL; t_max_ 60 min) and the intravenously (509.7 ± 20.5 ng/mL; t_max_ 60 min) administered formulae of free RT. These results demonstrated that chitosan-rivastigmine NPs enabled the direct transport of the API from nose to brain through the olfactory route and subsequently via the BBB. Further studies conducted by Wilson [34] also suggested the incorporation of RT in chitosan nanocarriers (by ionic gelation method), however, the authors only published data on the synthesis of the drug-loaded NPs and did not investigate their biological effects.

**Figure 3:**
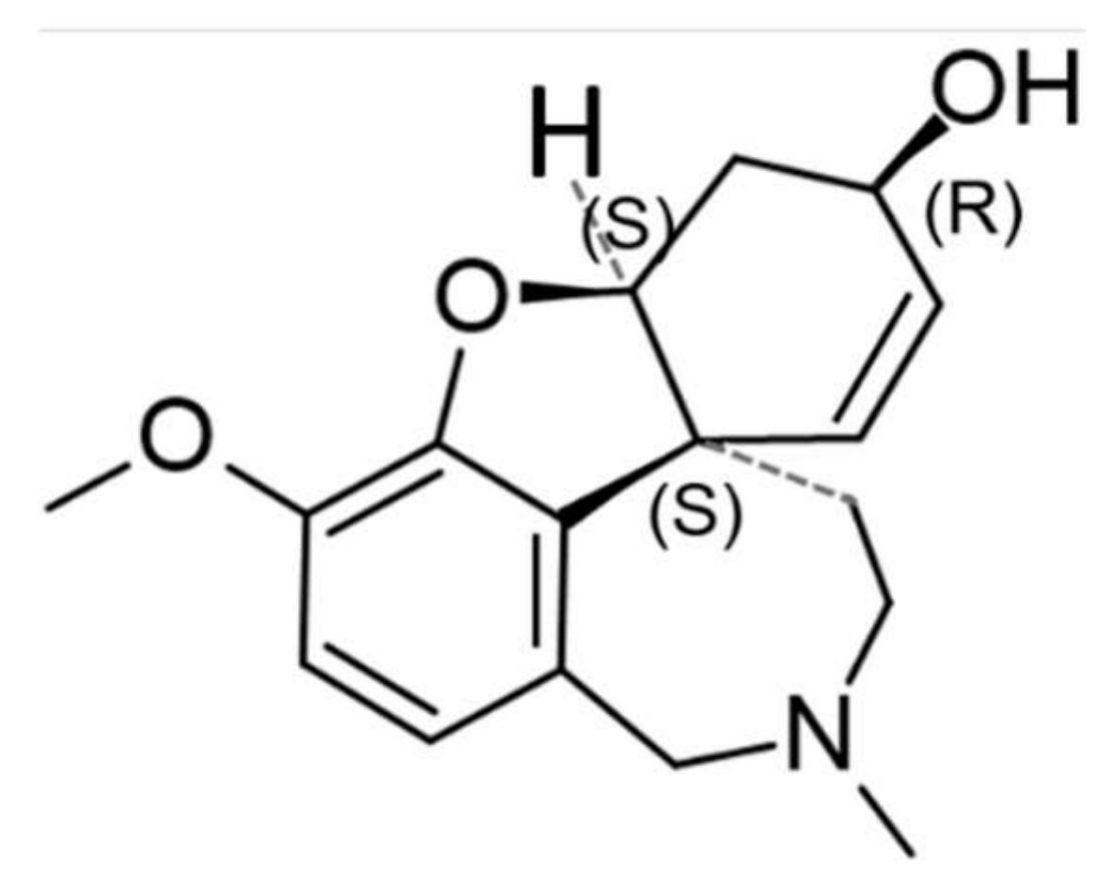
Chemical structure of galantamine.

Galantamine ((4a*S*,6*R*,8a*S*)-5,6,9,10,11,12-hexahydro-3-methoxy-11-methyl- 4a*H*-[1] benzofuro[3 a,3,2-ef][2] benzazepin- 6-ol) (GAL, Figure 3) is an FDA-approved, reversible and competitive AChE inhibitor [48]. Galantamine was also reported to enhance the sensitivity of nicotinic ACh acid receptors and prevent amyloid-beta aggregation. Albeit galantamine has a very high bioavailability (90–100%), both oral and injection-based administration causes severe side-effects, such as nausea, vomiting and muscle tremors which severely affect patient compliance [49].

With the intention of reducing side-effects of galantamine, Hanafy and coworkers encapsulated the API in chitosan NPs by inotropic gelation [42,50]. The chitosan-GAL nanoformula was administered to male Wistar rats intranasally (3 mg/kg/day). By applying fluorescent microscopic technique after labeling the drug loaded NPs with rhodamine B isothiocyanate, the authors demonstrated that chitosan-GAL NPs could reach multiple sections of the brain 1 h after i.n. administration; including the olfactory bulb, the hippocampus, as well as the orbitofrontal and parietal cortices. Within these regions, chitosan-GAL NPs were either located within intracellular vesicles – most probably lysosomes -, or free in the cytosol. The viability and intactness of neurons after administration of the loaded NPs indicated that their uptake was rather physiological – via the formation of endosomes – than due to increased membrane porosity or cell apoptosis. Detection of nanoparticles free in the cytosol also implied that the endosomes probably do not fuse with lysosomes and might release NPs directly in the cytoplasm, as it was hypothesized earlier [51]. Nanoformulation of GAL was also found to significantly enhance its AChE activity inhibiting effect in the brain compared to both orally and intranasally administered GAL solution applied at equivalent doses. In addition, chitosan-GAL NPs exerted their remarkable AChE protein reducing effect even 24 h after the last administration. Considering that GAL has a short half-life (~1.9 h) in rats, this implies that nanoencapsulation can prolong residence time of GAL, which can not only contribute to improved AChE inhibition, but also enable a less frequent dosing regimen.

**Figure 4:**
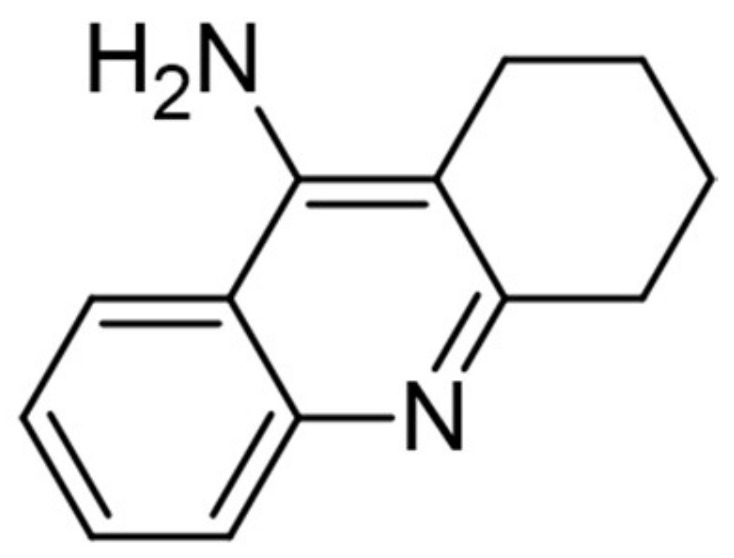
Chemical structure of tacrine.

Although tacrine (9-amino-1,2,3,4-tetrahydroacridine) (TAC, Figure 4) has been withdrawn from use for AD therapy, it was also encapsulated in chitosan nanoparticles in some studies in order to improve its physicochemical performance and brain accumulation. Tacrine is a non-selective acetylcholinesterase (AChE) inhibitor, which was the first molecule approved by the FDA for the symptomatic treatment of Alzheimer’s disease [52]. 

The applicability of tacrine was hindered by its very low bioavailability and its poor penetration through the BBB, which both contributed to the need for high and multiple doses [1]. In addition, use of tacrine was associated with cardiovascurlar as well as cholinergic (mainly gastrointestinal) adverse effects [53]. As tacrine also proved to be hepatotoxic—the single most important reason for drug withdrawal—, it was discontinued in 2013 [54]. Chitosan NPs may represent a potential solution to revive tacrine, by protecting the drug from excessive first-pass metabolism, enhancing its accumulation in the target brain regions and by provide means for its sustained release [1,3,28,30,40].

The therapeutic potential of chitosan-tacrine NPs (1 mg/kg, i.v.) was explored by Hassanzadeh et al. on male Wistar rats [2]. In their experiments, AD symptoms were generated by intracerebroventricular administration of streptozotocin (STZ) (3 mg/kg, i.v.). STZ is toxic to pancreatic beta cells and decreases glycogen synthase kinase (GSK-3) alpha/beta ratio (phosphorylated/total) in the brain. At the same time, intracerebroventricular or intraperitoneal administration of STZ increases the total amount of tau proteins and Aβ aggregation, enhances Aβ deposition and reduces cognition. Low dose administration of STZ in rodents was shown to generate neuroinflammation, oxidative stress and biochemical alterations, and is considered to be a reliable experimental model for diabetes and sporadic AD [55].

Hassanzadeh evaluated the effect of tacrine-loaded chitosan NPs on hippocampal-dependent spatial learning and memory was evaluated in Morris water maze tests. It was found that chitosan-TAC NPs could significantly decrease escape latency, reduce distance travelled and increase time spent on target quadrant, demonstrating their ability to prevent behavioral decline in AD. Chitosan-TAC NPs also largely reduced the expression of the amyloid precursor protein (APP), while induced the expression of the neuroprotective SELective Alzheimer Disease INdicator-1 (seladin-1) gene. It was also demonstrated that by incorporating magnetic particles in the nanocarriers, seladin-1 levels could be further elevated compared to the non-magnetic chitosan-TAC NPs. Based on these results the authors suggested that the magnetic approach is highly promising for future studies, as it not just enhances the BBB penetration of TAC, but is able to selectively deposit the drug into target brain regions contributing to even higher bioactivity at the site of action than non-magnetic chitosan NPs. Despite the favorable results, it has to be noted that the authors did not specify the details of nanoparticle synthesis and properties, neither compared the effects of chitosan-TAC NPs to that of the free drug or examined the peripheral side-effects of TAC-loaded chitosan nanocarriers. Chitosan was also suggested for the nanoencapsulation of TAC by Hassani et al., however, the authors did not research the bioactivity, pharmacokinetics or body distribution of the nanocarriers synthetized by ionotropic gelation [36]. Toxicity of chitosan-TAC NPs also produced by inotropic gelation was investigated in the studies of tamilselvan, where MTT (3-(4,5-dimethylthiazol-2-yl)-2,5-diphenyltetrazolium bromide) cell viability assays proved that tacrine-loaded chitosan NPs did not have toxic effects on SH-SY-5Y human neuroblastoma cell lines at 10 and 100 µg/mL concentrations, which implies their safety for further use in vivo [1]. In the studies of Wilson storage stability of chitosan-TAC NPs produced by spontaneous emulsification was confirmed: the drug-loaded NPs were both chemically and physically stable for at least 3 months [56].

### 2.2. Chitosan- Herbal Active Ingredient Nanoparticles

Aside traditional medications, medicinal plants have the potential to provide a complementary therapeutic approach for the treatment of Alzheimer’s disease [57]. However, most herbal active ingredients, especially lipophilic ones fail in clinical trials mainly due to low oral bioavailability, poor efficacy and insufficient safety. At the same time, it can be expected that—similarly to conventional drug compounds—these shortcomings can be addressed by the encapsulation of herbal ingredients in polymeric nanocarriers.

**Figure 5:**
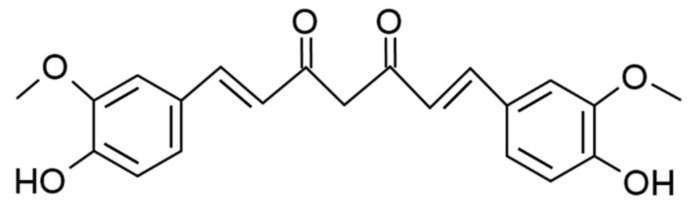
Chemical structure of curcumin.

Curcumin (CUR, (1*E*,6*E*)-1,7-bis(4-hydroxy-3-methoxyphenyl)-1,6-heptadiene-3,5-dione, Figure 5) is a well-known anti-inflammatory agent which was reported to have neuroprotective potential due to its anti-oxidative, metal-chelating and anti-amyloidogenic properties [58,59,60]. Curcumin also modulates macrophage polarization by inhibiting the toll-like receptor 4-mitogen-activated protein kinase (TLR4-MAPK)/NF-κB pathways [61]. On the other hand, curcumin exhibits very low water solubility, poor absorption by the intestines and high clearance rate, which greatly hinder its therapeutic potential [62]. The applicability of curcumin for brain disorders is further limited by its poor permeability via the BBB. In order to address these problems, incorporation of curcumin to chitosan NPs was suggested in several studies [63,64,65].

With the aim of enhancing curcumin-induced phagocytosis of the amyloid-β peptide, Yang prepared curcumin-loaded NPs by conjugating the positively charged chitosan with the negatively charged bovine serum albumin (BSA) [66]. Cellular uptake of CUR and chitosan-CUR NPs was investigated by confocal laser scanning microscopy on the macrophage cell line RAW 264.7. It was found that most of the free CUR was not taken up by the macrophage cells. In contrast, encapsulation in chitosan-BSA nanoparticles were found to greatly promote intercellular accumulation of CUR. In accordance with these results, enhanced phagocytosis of Aβ42 by RAW 264.7 macrophages was observed in the presence of CUR loaded NPs compared to macrophages treated with free CUR [67,68]. The authors suggested that chitosan-BSA nanocarriers can enhance the curcumin-induced macrophage phagocytosis by inhibiting M1 macrophage polarization via blocking the TLR4-MAPK/NF-κB signaling pathway. Transfer of various CUR formulations via the BBB was also evaluated in vitro by using monolayer transwell culture with the brain microvascular endothelial cell line hCMEC/D3, which is widely used to evaluate blood-brain barrier penetration of NPs. The model studies demonstrated that encapsulation in chitosan NPs significantly enhanced BBB transport of CUR compared to its free form, with 307%, 225% and 202% increase in its penetration rates at 1h, 2h and 3h, respectively. Endocytosis inhibition tests revealed that the variations in penetration rates can be explained by the difference in transport mechanisms of free and nanoformulated CUR. According to the authors, while free CUR can only pass though the BBB by passive diffusion, the transport of CUR NPs is an energy-dependent endocytotic process, which is primarily mediated by caveolae and macropinocytosis.

**Figure 6:**
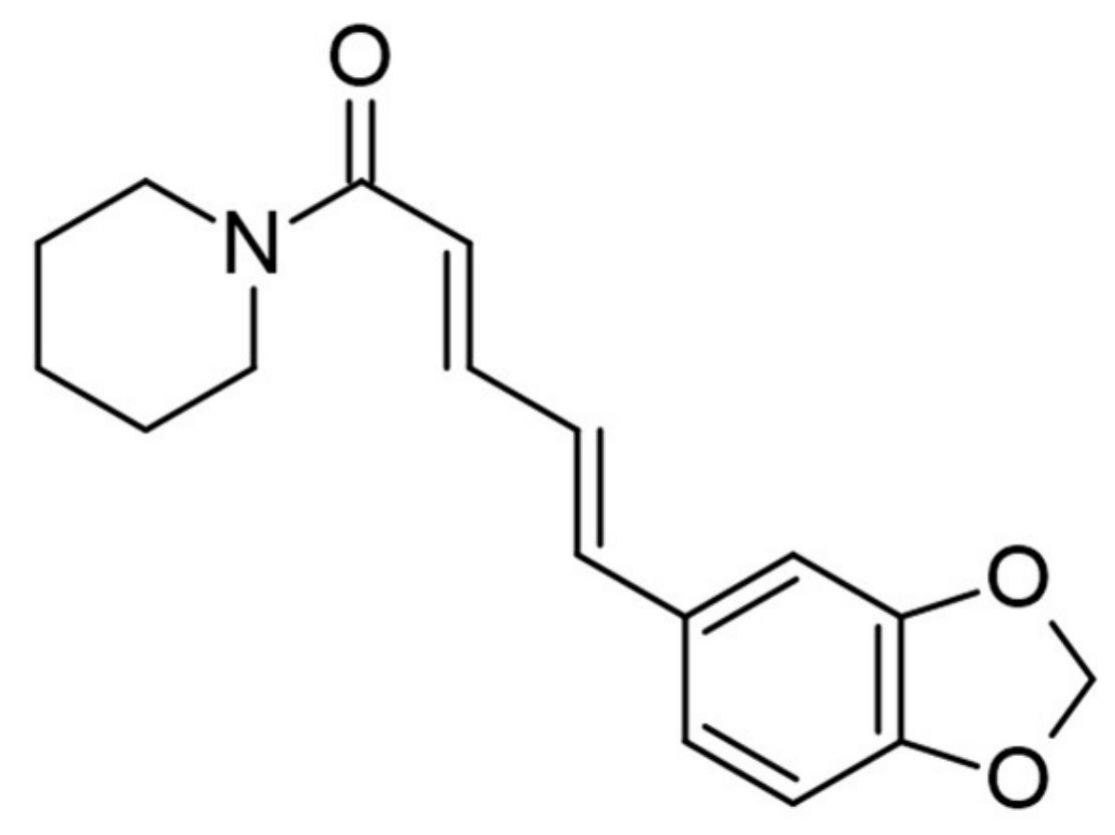
Chemical structure of piperine.

Piperine (PIP, (2*E*,4*E*)-5-(1,3-benzodioxol-5-yl)-1-piperidin-1-ylpenta-2,4-dien-1-one, Figure 6) is the primary pungent alkaloid of the plant *Piper nigrum*. Piperine has been used for centuries as a sternutatory agent, and possesses anti-oxidant, anti-inflammatory [67], analgesic [67], anticancer [68], anticonvulsant [69], antidepressant [70] and gastroprotective [71] effects. Piperine is also an agonist to the human capsaicin receptor TRVP1, which is a non-selective cation channel mainly expressed in the peripheral nervous system and of which activation is associated with the regulation of wide variety of biological responses including respiratory functions, body temperature, pain and glucose homeostasis [71,72]. Structure-activity relationship also suggests that due to its tertiary nitrogen (like in ACh), piperine can improve cognitive function in AD via the inhibition of AChE or by supporting the brain’s cholinergic system.

Piperine is subject to first-pass metabolism, however, intranasal administration can circumvent this problem [73]. At the same time, intranasal delivery comes with the disadvantage of very poor API bioavailability of 0.1% [74]. Furthermore, for nose-to-brain delivery of piperine, pungency of the compound has to be masked. Encapsulation of PIP in chitosan nanoparticles can provide solution for the above-mentioned problems, as well as offer further benefits in case of mucosal delivery due to the mucoadhesive nature of the polymer. The effect of intranasally administered, mucoadhesive piperine-loaded chitosan NPs on AD was investigated by Elnaggar on male Wistar rats [75]. Symptoms of AD were induced by centrally administered colchicine (i.v.), which is known to cause neurofibrillary degeneration, oxidative damage and increased AChE levels. Behavioral tests demonstrated that chitosan-PIP NPs significantly enhanced cognitive function and that their effect at 0.25 mg/kg/day (i.n.) dose was comparable to the effect of the standard drug donepezil administered at 2.5 mg/kg/day concentration intraperitoneally (i.p.). In comparison to the effective oral dose of free PIP, intranasally administered chitosan-PIP NPs enabled a 20-fold decrease of the effective dose of the active. Considering oxidative damage, only PIP NPs could improve substantially the state of oxidative stress, while the reference drug donepezil did not show any significant antioxidant potential. The fact that PIP NPs results were identical to that of the control group suggests that chitosan-encapsulated PIP may completely revert oxidative stress in AD. Nanoformulated piperine could also notably restore ACh activity, up to a level that equaled to the effect of donepezil. It is important to note that even though PIP NPs and donepezil showed the same efficacy in normalizing cognitive function and AChE levels, PIP NPs can be considered superior due to their dual mechanism. While donepezil only works on the cholinergic pathway, PIP NPs proved to be effective on two of the most important pathways in AD; both oxidative stress and the cholinergic pathway. From the formulation point of view, further benefit of chitosan encapsulation was that it effectively masked the pungency of piperine, thus it could be administered without any irritation of the nasal mucosa.

**Figure 7:**
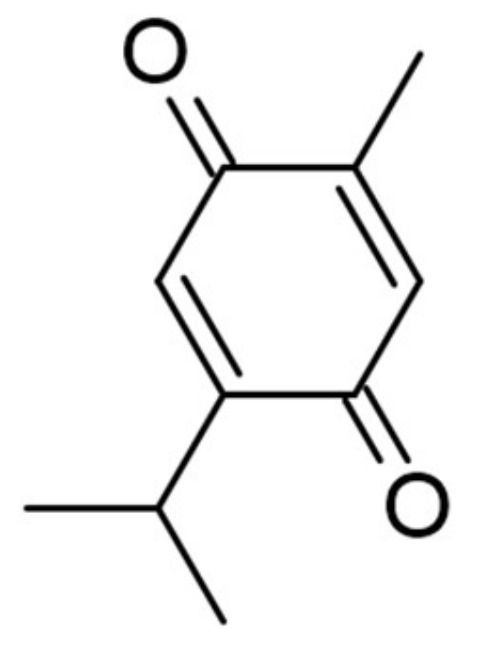
Chemical structure of thymoquinone.

Thymoquinone (TQ, 2-isopropyl-5-methylbenzo-1,4-quinone, Figure 7) is the major active component of the fennel flower (*Nigella sativa*) [76], which was reported to exhibit anti-inflammatory, antioxidant, analgesic and immunomodulatory effects, as well as the potential to ameliorate symptoms of neurodegeneration and cognitive deficits [77,78,79].

As TQ is hydrophobic and subject to hepatic first-pass metabolism, Alam and coworkers attempted to improve its pharmacokinetics and brain distribution by intranasally delivering the active with the help of chitosan nanocarriers. Biodistribution and pharmacokinetics of free and nanoformulated TQ was investigated in male Wistar rats (at 2.5 mg TQ/kg dose) by scintigraphic imaging after radiolabeling free drug molecules and NPs with ^99m^Tc. Both tissue concentration in brain and brain:blood ratio of TQ was higher in case of nanoencapsulated TQ than for the free TQ solution. Moreover, nanoparticles sustained TQ concentration in brain for 2–3 h, while in case of TQ solution no such phenomenon was observed. Enhanced nose-to-brain transport parameters also demonstrated the benefits of the mucoadhesive nanoformulation: upon encapsulation, 15-fold increase in brain-targeting efficacy (DTE%) and 2-fold increase in direct nose-to-brain transport (DTP%) was calculated from pharmacokinetic data, while relative bioavailability for NP-assisted delivery was 12.8 higher than for the conventional solution. The chitosan NP matrix was also found to enhance permeation of TQ via nasal mucosa ex vivo by 3-fold in 24 h compared to pure drug solution. While these results seem promising, it is important to note that TQ has been classified as a pan-assay interference compound (PAINS). As such, most of its biological activities are associated with its reactivity i.e., its redox activity and its ability to act as a Michael acceptor rather than noncovalent binding. In this manner TQ interacts with proteins in a nonspecific way, and thus often fail to show the desired pharmacological effect in clinical trials [80].

### 2.3. Chitosan NPs Combined with Other Therapeutic Agents

Aside AChE inhibitors and herbal active ingredients, other therapeutic agents, such as hormones, amyloid-β subfragments and hyaluronic acid were also combined with chitosan NPs to develop new therapeutics for the treatment of Alzheimer’s disease.

**Figure 8:**
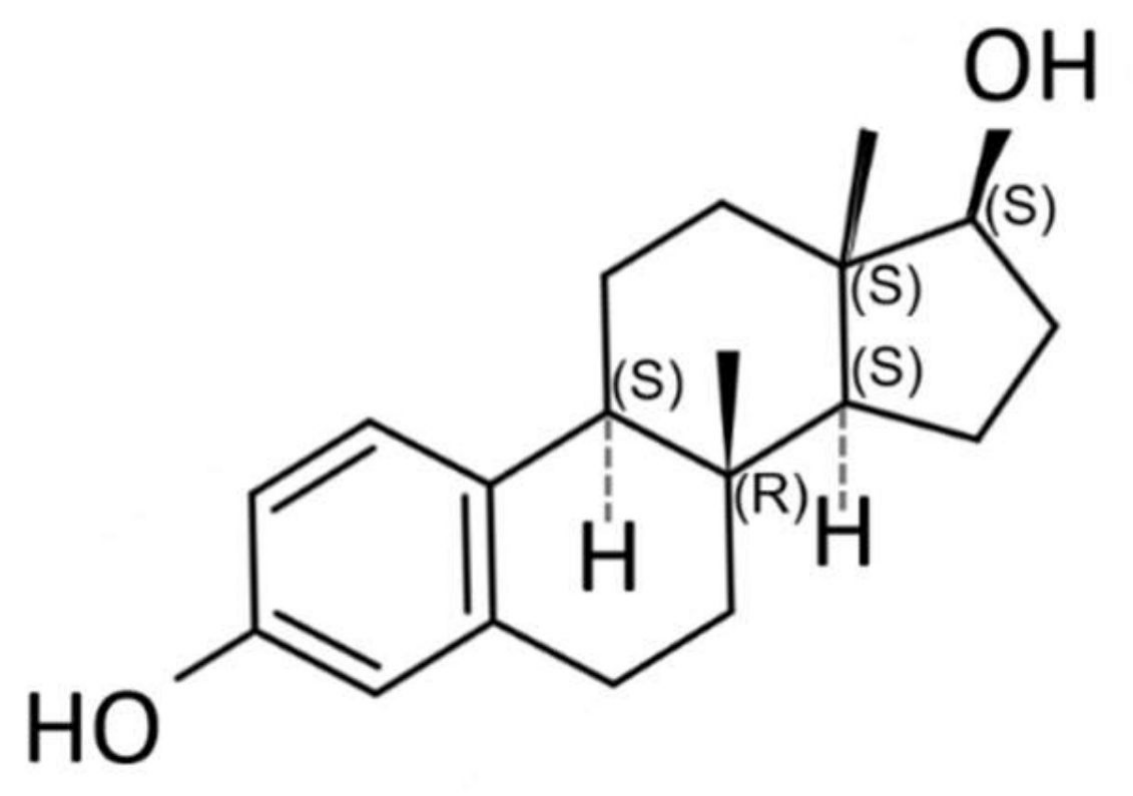
Chemical structure of 17β-estradiol.

17β-Estradiol ((8*R*,9*S*,13*S*,14*S*,17*S*)-13-methyl-6,7,8,9,11,12,14,15,16,17-decahydrocyclopenta [a]-phenanthrene-3,17-diol, E_2_, Figure 8) is a steroid-type female sex hormone, which is responsible for primary and secondary sexual characteristics. 17β-Estradiol is also associated with brain development, and proved to be useful in the prevention and amelioration of AD [81,82,83] However, similarly to most drugs, brain delivery efficacy of E_2_ after intranasal administration is limited by mucociliary clearance and the poor permeability of the nasal mucosa. In an attempt to improve its CNS deposition, Wang incorporated the hormone in chitosan nanoparticles [84]. Brain delivery after intranasal administration (0.48 mg/kg) was investigated by monitoring estradiol concentration in the cerebrospinal fluid (CSF) of male Wistar rats by in vivo microdialysis. Performance of chitosan- E_2_ NPs was evaluated in comparison to E_2_ inclusion complexes, i.e., formulations of estradiol with increased water solubility. It was found that chitosan NP-encapsulation resulted in higher E_2_ CSF concentrations at each sampling time as well as higher AUC_CSF_ values than the application of E_2_ inclusion complexes, proving that chitosan NPs are superior delivery systems for the brain targeting of estradiol.

Certain amyloid-β peptide subfragments were also found to show therapeutic efficacy on AD by effectively reducing amyloid deposition and having a toxic effect on inflammation antibodies in several studies [85,86,87,88]. In order to develop an Aβ subfragment formulation with enhanced BBB penetration and low immunogenicity, Songjiang and coworkers associated the 42-amino-acid amyloid-beta isoform Aβ42 (primary amino acid sequence: DAEFRHDSGYEVHHQKLVFFAEDVG SNKGAIIGLMVGGVVIA) with chitosan NPs [89]. CNS deposition of amyloid-β subfragment loaded chitosan NPs after i.p. administration (4.5–5.5 mg/kg) was examined on male Kunming mice by fluorescence spectrophotometry and fluorescent microscopy. It was found that nanoformulation drastically improved the brain uptake efficacy of the amyloid-β subfragment; from the original 20.7% to 80.6% in case of the nanoencapsulated peptide. Furthermore, ELISA assays confirmed that chitosan-amyloid-β NPs also exhibited favorable immunogenicity.

**Figure 9:**
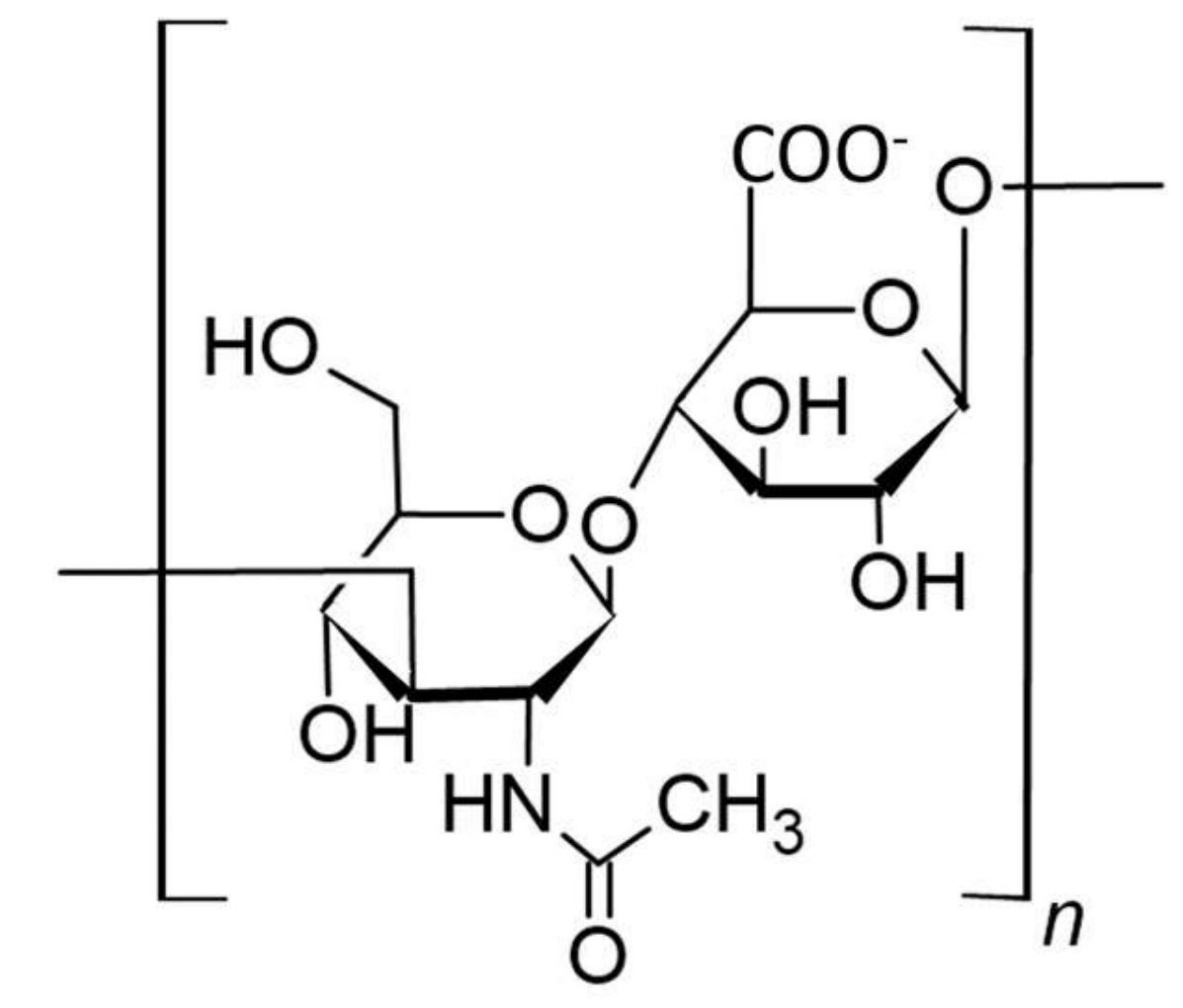
Chemical structure of hyaluronic acid.

Jiang et al. investigated the effect of polysaccharide nanoparticles on amyloid-beta aggregation by using chitosan-hyaluronic acid (HA) composite NPs [90]. As chitosan is positively, while HA (Figure 9) is negatively charged, they can form composite NPs in a self-assembling mechanism which is induced by electrostatic interactions, by the entropy gain due to the release of counterions as well as by the interactions via hydrogen bonds [91,92,93]. The surface charge of the composite NPs will be determined by the charge ratio of the anionic to cationic species. In the experiments of Jiang, seven types of stable chitosan-HA NPs were synthetized with the chitosan/HA mass ratio ranging between 9:1 and 1:15 [90]. The Zeta potential of the NPs changed in accordance with the mass proportions, varying from positive to negative by decreasing the chitosan/HA ratio. The authors demonstrated that all chitosan-HA NPs with a considerable surface charge could effectively hinder Aβ_40_ aggregation at physiological pH (7.4) based on thioflavin T fluorescent assays. In comparison, composite NPs with close to neutral Zeta potential as well as unformulated chitosan and unformulated HA did not have such an inhibiting effect. Generally, NPs with higher absolute Zeta potential values were more effective in hindering Aβ_40_ fibrillogenesis than those with lower absolute charges. Interestingly, it was also found that the largest positive Zeta potential (+35.8 mV) had a significantly higher inhibitory effect (61%) than that (45%) of the largest negative Zeta potential (−35.3 mV), even though their absolute values were very similar. It is important to note that these observations were independent from particle size. The detoxification effect of the composite NPs was also tested by using SH-SY5Y cell viability assays. After 20 h incubation at pH 7.4, Aβ_40_ aggregates decreased cell viability by 43%, while chitosan, HA and chitosan-HA NPs did not influence cell viability. It was found that all composite NPs with a measurable surface charge could ameliorate the toxic effect of amyloid-beta aggregates. Similarly to the Aβ_40_ plaque inhibitory experiments, the detoxification effect of NPs positively correlated with their absolute Zeta potential values. Positive charges were also found to be more effective, with the highest positive surface charge (+38.5 mV) contributing to 95% cell viability. The observed variations in the effect of NPs with different Zeta potential values were explained by investigating the secondary structures of protein aggregates by circular dichroism (CD) spectroscopy. Based on the CD spectra, positively and negatively charged NPs modified Aβ_40_ secondary structures differently: while positive NPs caused the lack of β-sheet elements, negative NPs generated a mixed conformation of α-helixes and β-sheets. NPs with very weak charge did not influence the protein structure significantly. Morphologic changes were further analyzed by atomic force microscopy (AFM). Aβ_40_ alone was found to form numerous mature fibrils with length in the micrometer range. Incubating positive NPs with Aβ_40_ decreased the number of these fibrils, while resulted in the size-increase of NPs, implying tight adsorption of protein fragments on the surface of nanoparticles. On the contrary, negative NPs could decrease the number of fibrils to a lesser extent. These observations can be explained by the differences in the electrostatic interactions of the NPs. Amyloid-beta has a net charge of −3.2, thus it can form stronger electrostatic interactions with positive NPs: positive NPs can bind to six acidic residues at the N-terminal of Aβ, contributing to the formation of larger and more stable complexes. In contrast, negative NPs can only bind to three positive residues on the protein which creates a weaker interaction compared to positive NPs.

**Figure 10:**
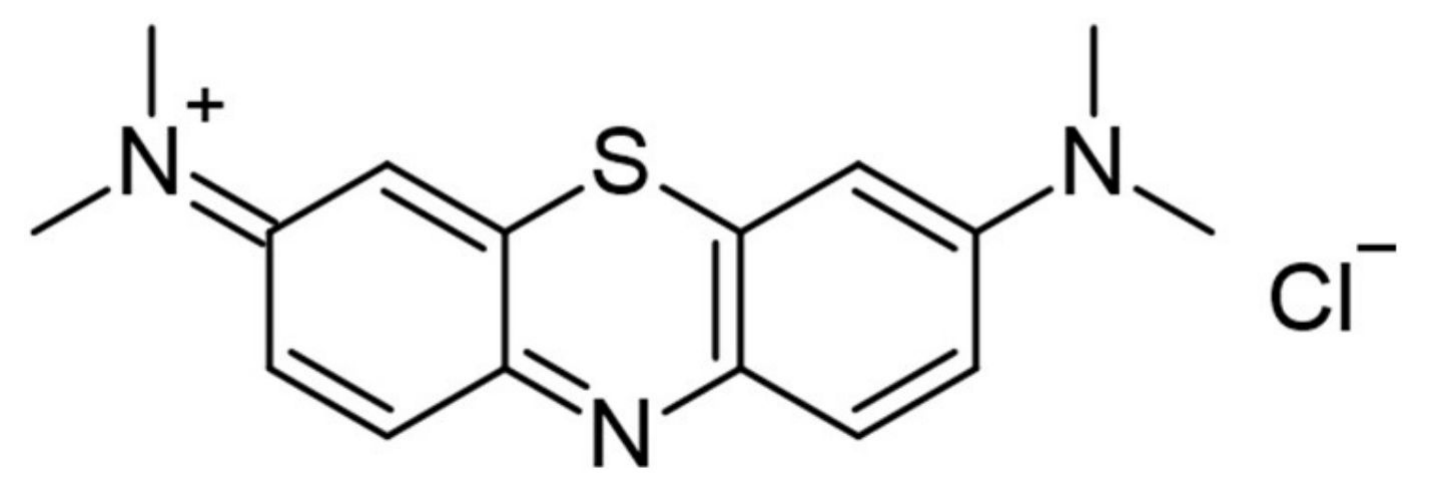
Chemical structure of methylene blue.

Methylene blue (3,7-bis(dimethylamino)phenothiazin-5-ium chloride) (MB, Figure 10) is a heterocyclic phenothiazine-class cationic die [94]. In its oxidized state, MB is water soluble and dark blue in color with the maximum adsorption at light wavelengths of 609 and 668 nm, while the reduced state of MB (leuco-MB) is uncharged, lipophilic and colorless. Oxidized MB and leuco-MB together form a reversible oxidation–reduction system or electron donor–acceptor couple [95,96]. MB was the first synthetic drug ever used in medicine for the treatment of malaria more than a hundred years ago [94]. MB was also the first synthetic compound ever used as an antiseptic in clinical therapy and the first antiseptic die that was applied therapeutically [95]. Currently MB is FDA-approved for the treatment of methemoglobinemia and is also employed for a vast array of other clinical indications, such as ifosfamide-induced encephalopathy; as redox coloring agent in biochemical studies; as stain in neuroanatomy, in bacteriology and for the anatomical visualization of tissues during surgery; as well as a targeting agent for various types of cancer [95,96,97]. MB has been also known to have beneficial effect on cognitive disorders for more than a century, since its utilization as one of the first APIs to address psychosis [94]. More recently, MB has been suggested to slow down cognitive decline in AD patients as it demonstrated to inhibit the accumulation of Aβ deposits and neurofibrillary tangles of tau proteins, to enhance tau degradation via autophagy and to decrease oxidative stress in vitro [96,98,99]. Certain neurotransmitter systems that are involved with the development of AD were also found to be influenced by MB, including cholinergic, serotonergic and glutamatergic systems [97]. In addition, MB was found to improve memory formation and cognitive function in rodents in vivo [100,101].

A remarkable benefit of MB for the treatment of neurodegenerative diseases is that, despite its high water solubility, it can cross the BBB as well as rapidly and extensively accumulate in the CNS after systemic administration [102,103]. Further advantages of MB are its relatively non-toxic nature, and that most of its side-effects are dose-dependent and do not occur with doses up to 2 mg/kg [98]. On the flip side, MB binds to and taken up by blood cells to a great extent, thus whole-blood measurements of MB may not correctly indicate its bio-phase concentrations [103,104,105,106]. MB is also subject to enzymatic reduction either by NADH/NADPH dehydrogenases or reductase enzymes in red blood cells and peripheral cells to leuco-MB, which limits its applicability both for therapeutic and imaging purposes [107]. In addition, in therapeutic applications MB causes the discoloration of skin, urine, mucous membranes and sclera, which can be alarming to patients and reduce patient compliance [94]. Despite promising in vitro and in vivo preliminary results, hydroxymethylthionine, the stabilized, reduced form of MB currently investigated in clinical trials, also failed to attenuate the progression of AD in the latest phase III randomized controlled trials and was simultaneously found to provoke urinary and gastrointestinal side effects [108,109]. In order to address these above-mentioned deficiencies, development of alternative systems for the delivery of MB are required.

The incorporation of MB in biodegradable polymeric nanocarriers was investigated in several studies as a means to potentially improve bioavailability and patient compliance; provide controlled release; and to protect the die from enzymatic reduction and thus reduce required doses [107,110,111]. Some authors also suggested the incorporation of MB in polymeric NPs with the aim of potentially enhance its neuroprotective [110], anti-Alzheimer [112] and antitumor effect on glioblastoma [113,114,115,116]. Among these, Castañeda-Gill demonstrated that encapsulation of MB in PLGA NPs coated with Pluronic F68 (polyoxyethylene-polyoxypropylene-polyoxyethylene triblock copolymer) increased the BBB penetration ability of MB by 60% [117]. In the reports of Jinwal and Grover in vitro BBB Transwell™ studies confirmed that the glutathione-coated PLGA nano-encapsulated MB had more than 6.5-times higher permeation via a co-culture of RBE4 rat brain endothelial and C6 astrocytoma cells than the conventional MB solution 24 h after administration [112,114]. It was also found that the polymeric NP encapsulated MB effectively reduced both endogenous and over-expressed (by transfected HeLa cells) tau protein levels in human neuroblastoma SHSY-5Y cells. Furthermore, polymeric NPs enabled the sustained release of MB over the course of 144 h [112]. While no studies have been published on the brain delivery of MB with the help of chitosan NPs, chitosan was shown to have a high adsorption capacity for a vast array of dies and is widely reported as a matrix material for MB adsorption, e.g., from wastewater [115,116,117,118]. In some other studies chitosan NP-MB formulations were developed as model drug delivery systems [119,120]. Chitosan was also investigated in combination with biodegradable and non-biodegradable additives as a carrier enabling the controlled release of MB governed by external stimuli, such as pH [121,122], temperature [123] and ionic strength [122,124]. Such systems could open new possibilities in the CNS delivery of MB such as keeping drug concentration in the desired range, lowering frequency of administration, or reducing side-effects and toxicity [125]. Here we have to note that as MB is cationic, low loading efficacy in chitosan-based NPs can be expected due to electrostatic repulsion with the positively charged polymer, which contradicts the observed high adsorption capacity of chitosan towards MB. This discrepancy can be explained by that in crosslinked chitosan networks (such as gels and nanoparticles) the presence of negatively charged crosslinker moieties covalently bound to the chitosan backbone can mediate local electrostatic complexation between the polymer and the positively charged groups of MB. Therefore, by carefully selecting crosslinkers during the synthesis of chitosan NPs the network electrostatic charge can be systematically tuned to promote chitosan-MB electrostatic complexation, thus an almost complete MB loading can be achieved [120]. Based on these findings, chitosan NPs can be suggested as delivery vehicles to improve performance of MB in the treatment of AD.

Another interesting prospect in the treatment of AD can be the combination of MB loaded chitosan NPs with photodynamic therapy (PDT). Light-induced inhibition of Aβ aggregation by utilizing photosensitizing agents including MB has recently been suggested [126,127]. MB is well-known photosensitizer which is extensively used in PDT for the treatment of cancer cells and microbes due to its high quantum yield (ΦΔ ~ 0.5) of singlet oxygen (^1^O_2_) upon red light irradiation (>630 nm). Photo-excited MB molecules were found to exhibit significantly higher inhibitory effect on the fibrillogenesis and on the toxicity of Aβ42 than static MB both in vivo and in vitro, due to their oxidizing effect via the generation of ^1^O_2_ [108]. As tissue penetration of red light is better than that of green or blue light, MB also has an advantage over other anti-amyloid photosensitizers (such as metal oxides or organic compounds), which can be only excited by lower wavelengths. In addition, MB is cheaper and more easily available than conventional photosensitizers [128]. However, as mentioned earlier, in case of systemic administration MB is subject to quick reduction to its leuco-MB form, which significantly hinders its clinical applicability for PDT due to the negligible photodynamic activity of leuco-MB [129,130]. As encapsulation in polymeric NPs can provide solution to this problem [129,131], several authors suggested the incorporation of MB in chitosan NPs with the aim of protecting the die from enzymatic reduction and enhancing its efficacy in PDT treatments [128,132,133,134,135,136,137,138], which improvements could be utilized in the treatment of AD as well. Nonetheless, it also has to be noted that delivery of light into brain tissues through the skull represents a major obstacle in the applicability of PDT for AD therapy. However, recent advances in optogenetics, such as transferring light into deeper brain regions with the help of optic fibers or the development of wireless implantable microLED devices [108] may open possibilities for the application of phototherapies utilizing MB-loaded chitosan NPs for neurodegenerative disorders in the future.

## 3. Discussion

In the last decade, numerous studies suggested chitosan NPs as delivery systems of a wide variety of anti-Alzheimer agents including acetylcholinesterase inhibitors (such as tacrine, rivastigmine and galantamine hydrobromide), herbal active ingredients (e.g., curcumin, piperine, thymoquinone), as well as estradiol, amyloid-beta subfragments and hyaluronic acid (Table 1). In all of these studies, chitosan NP-mediated delivery proved to be superior to conventional formulations in accumulating anti-Alzheimer therapeutics in the brain and ameliorating the symptoms of AD. In addition, it was found that administration of AD drugs in combination with chitosan nanocarriers can also contribute to an array of other advancements; such as enhanced antioxidant effect [75], improved inhibition of cholinesterases [2,42], increased half-life [42], lower effective doses [75], reduced toxicity [44] and masked pungency of actives during intranasal dosing [75]. While these improvements demonstrate the high potential of chitosan NP in addressing the symptoms of AD, in order to design clinically-applicable chitosan NP-based Alzheimer disease therapeutics, relationship between composition, physicochemical properties and therapeutic performance of NPs have to be considered in a more comprehensive manner.

Firstly, molecular weight of chitosan is crucial in the development of successful nanoformulae for the treatment of AD. High molecular weight chitosan is characterized by low solubility in aqueous media as well as high viscosity in solution [39], which hinder its applicability as a drug nanocarrier. As lowering the molecular weight of chitosan proportionally increases its water solubility [139], application of low Mw chitosan is suggested for the development of drug nanoformulae. It was also reported that by decreasing chitosan molecular weight, higher drug encapsulation efficacy can be obtained [140]. Furthermore, lower Mw (preferably combined with a deacetylation degree lower than 70%) is more beneficial in enhancing biodegradation rate of chitosan NPs [141,142,143]. The molecular weight of the polymer is pivotal for biodegradability and toxicity too, as nanocarriers based on low Mw chitosan generally show higher degradation rates and lower cytotoxicity compared to higher Mw forms [141,142,143]. Furthermore, the lower the molecular weight, the higher inherent antioxidant activity chitosan exhibits [144], which is particularly beneficial in case if anti-Alzheimer nanosystems.

**Figure 11:**
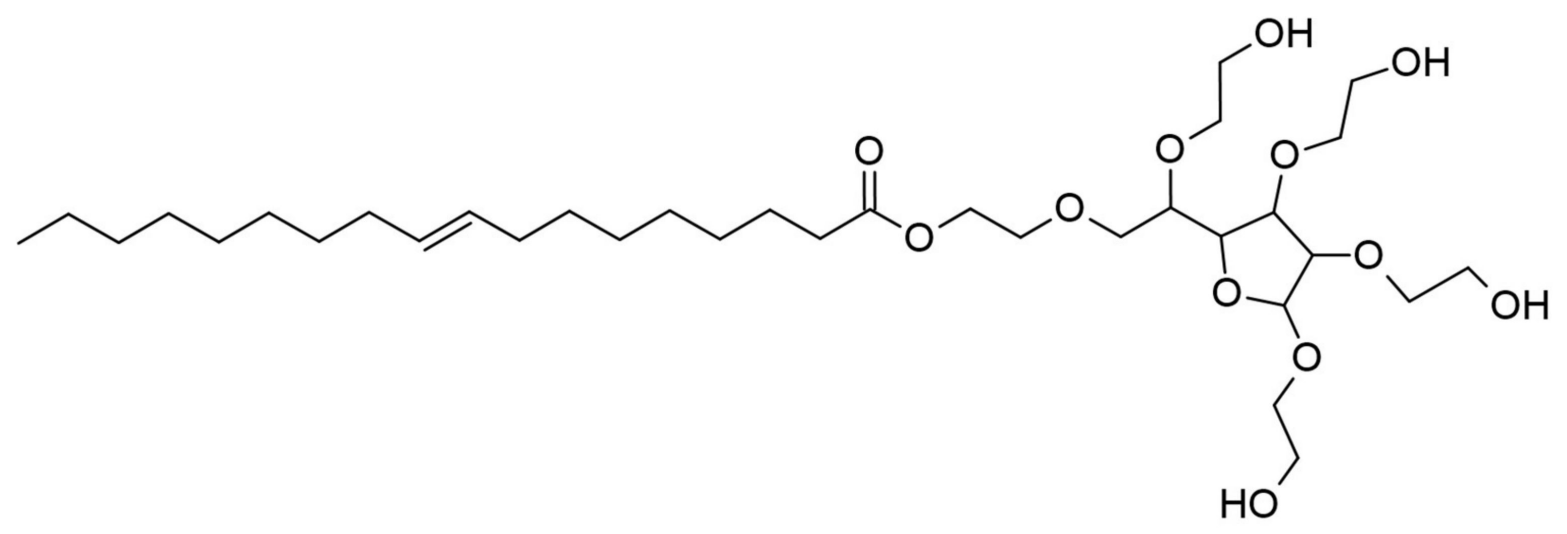
Chemical structure of polysorbate 80.

Secondly, effects of nanoparticle surface coating and charge have to be considered. Several studies reported that coating drug-loaded NPs with polysorbate 80 (Figure 11) can change both their pharmacokinetics and pharmacodynamics [145,146,147].

Probably the most important benefit of coating chitosan NPs with polysorbate 80 is that it can enhance brain accumulation of the carried drugs [41,42,132]. This improved targeting is probably attributed to the preferential binding of polysorbate 80 coated NPs to serum apolipoprotein (Apo-E). According to the theory, these Apo-E bound NPs are able to mimic low density lipoproteins (LDL) and thus bind to the LDL receptors on the microvascular endothelial cells of the brain [148]. As a result, polysorbate 80 coated nanocarriers are endocytosed, then subsequently transcytosed through the endothelium, and eventually are re-uptaken by neurons into the brain parenchyma [133,149]. It was also found that uptake of rivastigmine-, as well as tacrine-loaded chitosan NPs by non-target organs (i.e., liver, spleen, heart and lung) could be reduced drastically by coating the nanocarriers with polysorbate 80 [29,40], which can prove particularly useful in the reduction of side-effects of Alzheimer drugs. Further advantages of polysorbate 80 coating were also demonstrated in several studies, such as increased MTD [38,42]; decreased opsonization and phagocytic uptake by Sertoli cells; inhibition of the P-glycoprotein efflux system [150,151]; lower initial burst effect during drug release [34]; as well as reduced biochemical and hematological adverse effects [44]. Based on these results the potential improvements outweigh the possible negative effects of polysorbate 80 coating—i.e., a moderate decrease in the cumulative percentage release of APIs [34]—, thus surface coating of chitosan nanocarriers should be considered for AD therapeutic purposes.

Surface charge of chitosan NPs is also a pivotal property that can determine their therapeutic success. Chitosan exhibits a cationic nature due to the presence of amine groups at the C6 position of its pyranose ring, which can be both beneficial and disadvantageous in biomedical applications. On one hand, this positive charge can be employed for the facile preparation of chitosan NPs, for instance by self-assembly [35,90] or by ionotropic gelation [36,44,47]. The hydrophilic and cationic amino groups also support mucoadhesivity of the polymer by electrostatically binding to the negatively charged sialic acid moiety of the mucosa. Furthermore, positive charge of chitosan NPs was also reported to contribute to their cellular uptake via electrostatic interactions with negatively charged cell surfaces as well as to their aqueous dispersibility [40]. At the same time, the fact that PEG-modified chitosan NPs were found to be less harmful on cells in MTT assays than uncoated chitosan NPs may indicate that excess positive charge attributed to free amine groups could disturb the integrity of cell membranes up to the point of provoking cell death [43]. The cationic quality of chitosan can also limit the encapsulation efficacy in case of cationic drugs [42]. Surface charge also plays a crucial role in the stability of chitosan-based drug nanocarriers, as electrostatic repulsion between individual particles greatly affects their dispersion state. NPs with negative surface charge were found to show poor aggregation stability which is attributed to the weak electrostatic repulsive forces between individual particles [152]. As a result, these NPs are subject to rapid aggregation and a subsequent clearance from the blood by the macrophages of the RES [153]. However, API nanocarriers with high positive surface charges are also disadvantageous: on one hand they are more effectively phagocytized than NPs with smaller positive charges [25], and on the other hand, they were shown to have immediate toxic effect on the BBB [154]. For that reason, nanoparticles for the treatment of AD should possess small positive surface charge in order to maximize their circulation time in the blood as well as to avoid BBB toxicity [24,25]. Lastly, surface charge of chitosan NPs has a pronounced effect on Aβ aggregation too [77,143,144], however, controversial results have been published on the exact impact of positive and negative charges. While certain studies implied that positively charged NPs can inhibit Aβ fibrillogenesis [155,156,157], in others their Aβ aggregation stimulating effect was reported [158,159]. Similarly, negative charges were both suggested to suppress [160,161] and to promote Aβ fibrillogenesis [162,163]. Most probably the amyloid-beta aggregation inhibiting or promoting effect of NPs is a highly complex question which depends on a number of physicochemical factors, such as surface charge, hydrophobicity, pH as well as on the type of Aβ species [164,165].

Stability of the nanocarriers as well as the encapsulated APIs is a deciding factor in therapeutic efficacy too, however, it was investigated only in a few studies. In the reports of Wilson et al., it was demonstrated that both rivastigmine- and tacrine-loaded chitosan NPs showed sufficient chemical and physical stability for at least 3 months, retaining their pharmaceutical properties at room temperature (15–20 °C), as well as at low (3–5 °C) and elevated (37 °C) temperatures [34,56]. In contrast, Hanafy observed that while galantamine-loaded chitosan NPs showed sufficient chemical and physical stability at 4 °C, significant drug leakage, as well as increase in particle size and polydispersity occurred when the nanocarriers were stored at 25 °C in their aqueous dispersion [42]. Hassani emphasized that chitosan nanocarriers prepared by inotropic gelation method are not stable in neutral media, therefore acidic pH is required to ensure their integrity during long-term storage [36]. This phenomenon was explained by the sensitivity of chitosan’s protonation degree to solution pH: above a critical pH value chitosan becomes less protonated, which decreases its cross-linking capacity and thus destabilizes its nanoparticles [166]. API stability within the polymeric matrix was also evaluated occasionally by differential scanning calorimetry (DSC) and by X-ray powder diffraction analysis [38,47,76]. These studies reported that recrystallisation of the loading drug molecules - such as RT and TQ - during the encapsulation in chitosan NPs could be avoided, and the API were successfully stabilized either as molecular dispersion or amorphous dispersion. Such stabilization effect is highly desirable in the development of novel AD medications, as it can contribute to significant increase in water solubility of hydrophobic drugs as well as to enhanced bioavailability [167].

Similarly, the drug release profile of chitosan NP-based anti-Alzheimer medications has to be assessed carefully. Chitosan nanocarriers commonly show a biphasic drug release pattern. First, an initial burst discharge happens, where API molecules adsorbed on the surface layer and entrapped within the pores of the nanocarrier are quickly released to the surrounding media. In the second stage, a lag phase and a zero-order phase follow, which is attributed to the diffusion of API molecules across the polymeric network, as well as to the degradation of the chitosan matrix [34,168]. Due to the gradual nature of the second stage, if no drug discharge inhibiting interactions (e.g., chemical bonding) are present between the API molecules and the polymer matrix, most chitosan-based nanocarriers enable sustained drug release, which can greatly contribute to the reduction of peripheral side-effects of anti-Alzheimer drugs. In accordance with this theory, a biphasic drug release profile comprising of an initial burst discharge and a subsequent sustained release phase was observed in all studies reviewed in this manuscript, however, duration of the phases and the amount of discharged drug molecules varied considerably. For instance, Fazil and Nagpal found that after the initial burst discharge, RT molecules were released form their chitosan nanocarriers in a sustained manner over the period of 24 h [38,44]. In contrast, Wilson reported that chitosan-based NPs were able to obtain sustained release of RT only for 12 h [34]. Likewise, different sustained release durations were observed in case of tacrine nanocarriers; from 12 h [56] to 24 h [1]. Among cholinesterase inhibitors, galantamine hydrobromide showed the longest sustained release period, which extended over 72 h [42]. The variations in the drug release kinetics of ChE inhibitors can be explained by specific interactions between the APIs and the polymeric matrix [169]. For tacrine and rivastigmine, lack of interaction with the chitosan matrix was confirmed by FT-IR analysis [34,56]. On the contrary, in case of galantamine, FT-IR and DSC spectra as well as the exceptionally prolonged drug release period (72 h) implied the formation of hydrogen bonds between the API and the polymer chains [42]. Similarly to AChE inhibitors, herbal active ingredients such as curcumin [66], thymoquinone [76] and piperine [75] were also found to be released in a sustained manner after a short burst discharge, usually over the course of 24 h.

As the brain has very limited regenerative capacity [170], potential toxicity of chitosan nanoparticle-based anti-Alzheimer therapeutics also has to be scrutinized before their clinical application, however, it was only investigated in a few experiments. For example, safety of curcumin loaded chitosan-BSA NPs was confirmed by using cell viability MTT assays with RAW 264.7 cells (M1) and hCMEC/D3 [66]. In case of piperine loaded chitosan NPs, potential toxicity was evaluated by assessing neural apoptosis in caspase-3 assays and by monitoring neural inflammatory reaction by measuring TNF-α levels in the hippocampus [75]. Both methods proved that the nanotherapeutics were non-toxic, moreover, they were shown to exhibit anti-apoptotic and anti-inflammatory effects [75]. Chitosan NPs were also found to be safe for intranasal delivery [171], which is of utmost importance as nasal administration represents a high potential, yet simple and non-invasive way to circumvent the BBB [172]. For example, it was confirmed that intranasal administration of galantamine NPs did not lead to histopathological manifestations in the olfactory-bulb, neither in other brain regions including the orbitofrontal cortex, parietal cortex or hippocampus. Furthermore, orbitofrontal cortex neurons also exhibited intact nuclear membrane, mitochondria as well as endoplasmic reticulum after the uptake of chitosan-GAL NPs [42,50]. Aside using biodegradable and biocompatible polymers to build nanocarriers of AD therapeutics, special attention has to be paid to synthetize the NPs without using toxic additives, surfactants or solvents. For instance, chitosan NPs were formed by emulsification in some studies using toluene [34,56], which is a well-known neurotoxin that can contribute to cognitive impairment including dementia [173]. Another problem is the general use of potentially harmful crosslinking agents, such as glutaraldehyde [44,56,145], which both found to have adverse effects on various organs in animal as well as in human studies [174]. Therefore, the use of safer crosslinking methods in the future development of chitosan NP-based AD medications is highly important. Such options can be crosslinking with the help of sulfuric acid and heat treatment, or exploiting chitosan’s inherent tendency towards self-assembly. At the same time, effect of the type of crosslinker on the biodegradation rate of chitosan NPs should not be neglected either, as certain crosslinkers were found to contribute to faster degradation (e.g., glutaraldehyde) than others (e.g., tripolyphosphate) [175].

While the above reviewed improvements might be achieved by using other biodegradable nanopolymers too, chitosan emerges as the probably most promising material for the treatment of Alzheimer’s disease due to its mucoadhesive as well as its inherent bioactive nature. Intranasal administration of AD drugs is highly advantageous, as it is non-invasive, painless, does not require sterile preparations and allows the patients to dose themselves [176]. Furthermore, it is characterized by a fast onset of action, helps with bypassing presystemic metabolism, and enables the reduction of side-effects [172]. Intranasal administration also facilitates direct and non-invasive brain delivery as it can target the olfactory nerve system, which is the only exposed part of the CNS [177]. However, delivery of anti-Alzheimer therapeutics via the olfactory route is limited by a number of factors. One of them is the mucociliary clearance, which rapidly removes intranasally administered formulations in normal circumstances and thus largely reduces the timeframe for drug absorption as well as disables sustained drug administration [38]. The other major restriction is the low permeability of the nasal mucosa for drug molecules, which raises the need for penetration enhancing formulations. The mucoadhesive chitosan NPs can provide effective solution for both problems, by delaying mucociliary clearance and simultaneously enhancing drug transport via the BBB [178]. In accordance with this theory, benefits of using chitosan NPs for the intranasal administration of anti-Alzheimer drugs were demonstrated in some of the reviewed studies. In case of rivastigmine loaded chitosan NPs, nasal mucosa permeation enhancing effect of chitosan NPs was shown [38]. This improvement was explained by the ability of chitosan NPs to transiently open the tight junctions of mucosal endothelial cells by interacting with the negatively charged sites of cell membranes via its positively charged amino groups [179]. It was also found that encapsulation of piperine in chitosan NPs facilitated the formation of clear solutions form the normally water-insoluble active ingredient, and thus enabled its intranasal dosing without clogging the olfactory bulbs [75]. In addition, chitosan NPs also effectively masked the pungency of piperine so it could be administered without irritation of the mucosa. The other unique characteristic of chitosan compared to other biodegradable polymers is that it can act as a therapeutic agent itself in the treatment of neurodegenerative diseases, including AD [180,181,182]. In the last decade several studies demonstrated the potential of chitosan, as well as chitooligosaccharides and their derivatives to improve the symptoms of AD via a number of mechanisms, such as:

- Fighting neuroinflammation by suppressing lipopolysaccharide-induced macrophage responses via the MAPK signaling pathway, as well as by inhibiting the activation of neutrophils, basophils and lymphocytes [183,184,185,186].
- Scavenging superoxide free radicals by combining with them through free hydroxyl and amino groups [187].
- Inhibiting β-secretase production [188], hindering Aβ agglomeration [189] and preventing Aβ-induced cell apoptosis [190].
- Inhibiting acetylcholinesterase [191].
- Alleviating metal ion dynamic equilibrium disorder by chelating copper ions [192].
- Promoting neuronal differentiation, synaptic growth and nerve cell adhesion [193], as well as reducing neuronal apoptosis induced by glutamate [194].

However, it is important to note that though chitosan NPs are proved to be able to hinder Aβ aggregation and its negative consequences, designing effective cerebrovascular amyloid deposit targeting nano-vehicles is highly challenging. In order to successfully target these therapeutic nanovehicles, they have to fulfil several criteria at the same time. First of all, NPs have to be water-dispersible and possess high colloidal stability. Second, they have to be fully able to transport via the BBB. Once in the brain, their retention time in the brain vasculature has to be sufficiently long, without diffusing into the brain parenchyma. Lastly, NPs have to successfully recognize and bind to amyloid-beta deposits.

The findings summarized in present manuscript clearly demonstrate that chitosan is not just a high potential carrier for the nanoencapsulation and BBB delivery of anti-Alzheimer drugs, but it can also act as a therapeutic agent on its own to prevent and improve the symptoms of Alzheimer’s disease. As currently there is a growing interest in exploring natural bioactive compounds for the treatment of neurodegenerative disorders [3], chitosan nanoparticles can undoubtedly be proposed as subjects of future studies aiming to overcome current challenges in the management of AD.

## Figures and Tables

**Table 1:** Overview of chitosan-based nanoparticles developed for the treatment of Alzheimer’s disease.

API	Additive	NP Synthesis Method	NP Size (nm)	NP Morphology	Encapsulation Efficacy *	References
rivastigmine	-	ionotropic gelation	154	spherical	96%	Nagpal et al. [44]
	-	ionotropic gelation	44	subspherical	85%	Khemariya et al. [47]
	-	ionotropic gelation	184	spherical to subspherical	86%	Fazil et al. [38]
	-	spontaneous emulsification	47	not specified	74%	Wilson et al. [34]
galantamine	-	ionotropic gelation	182–190	spherical to subspherical	23%	Hanafy et al. [42,50]
tacrine	-	not specified	not specified	not specified	not specified	Hassanzadeh et al. [2]
	-	ionotropic gelation	50	sub-spherical	42–72%	Tamilselvan et al. [1]
	-	spontaneous emulsification	41	spherical	80%	Wilson et al. [56]
	-	ionotropic gelation	90–100	not specified	65%	Hassani et al. [36]
curcumin	BSA	electrostatic interaction-based conjugation	145	spherical	95%	Yang et al. [66]
piperine	-	ionotropic gelation	249	spherical	82%	Elnaggar et al. [75]
thymoquinone	-	ionotropic gelation	172	spherical with smooth surface	63%	Alam et al. [76]
17β-estradiol	-	ionotropic gelation	270	not specified	65%	Wang et al. [84]
Aβ subfragments	-	combined emulsification and crosslinking	15	spherical	79%	Songjiang et al. [89]
no cargo	HA	electrostatic interaction induced self-assembly	67–698	not specified	-	Jiang et al. [90]

* Percentage of drug that is successfully entrapped in the nanoparticle calculated by (total drug added – free non-entrapped drug) divided by the total drug added.
